# Access to mobile phone, socio-economic equity and maternal and child healthcare utilization in Rwanda: analysis of demographic and health surveys

**DOI:** 10.1093/oodh/oqaf018

**Published:** 2025-08-14

**Authors:** Amare Tariku, Betelhem Abebe Andargie, Tesfahun Melese Yilma, Abdulaziz Mohammed, Admas Abera, Diwakar Mohan, Shivani Pandya, Lena Kan, Hinda Ruton, Meredith Kimball, Patricia Mechael, Smisha Agarwal, Binyam Tilahun

**Affiliations:** Center for Digital Health and Implementation Sciences, University of Gondar, Gondar, Ethiopia; Center for Digital Health and Implementation Sciences, University of Gondar, Gondar, Ethiopia; Center for Digital Health and Implementation Sciences, University of Gondar, Gondar, Ethiopia; Center for Digital Health and Implementation Sciences, University of Gondar, Gondar, Ethiopia; Center for Digital Health and Implementation Sciences, University of Gondar, Gondar, Ethiopia; Johns Hopkins Bloomberg School of Public Health, Johns Hopkins University, Baltimore, MD, United States; Johns Hopkins Bloomberg School of Public Health, Johns Hopkins University, Baltimore, MD, United States; Johns Hopkins Bloomberg School of Public Health, Johns Hopkins University, Baltimore, MD, United States; Africa quantitative sciences, Rwanda; Exemplars in Global Health, Gates Ventures, Seattle, WA, United States; Johns Hopkins Bloomberg School of Public Health, Johns Hopkins University, Baltimore, MD, United States; Johns Hopkins Bloomberg School of Public Health, Johns Hopkins University, Baltimore, MD, United States; Center for Digital Health and Implementation Sciences, University of Gondar, Gondar, Ethiopia

**Keywords:** access to mobile phone, maternal and child health, socio-economic equity, digital health, demographic and health survey, Rwanda

## Abstract

Rwanda is harnessing digital health as a key strategy to improve optimal access to quality maternal and child health services, aiming to reduce maternal and child mortality and attain sustainable development goals. Evidence is essential to guide Rwanda’s effort to integrate digital health technologies with maternal and child health service delivery. This study analyzed data from 2010, 2014/15 and 2019/20 Rwanda demographic and health surveys to explore trends and socio-economic equity in mobile phone ownership and its influence on reproductive and maternal health service utilization. Results showed a marked increase in household mobile phone ownership from 40% in 2010 to 71% in 2019/20. Significant pro-rich inequities in household mobile phone ownership were observed in 2010 (concentration index = 0.46), 2014/15 (concentration index = 0.28) and 2019/20 (concentration index = 0.22). Mobile phone was also higher among the educated in 2010 (slope index = 0.63), 2014/15 (slope index = 0.59), and 2019/20 (slope index = 0.50). Mobile phones were inequitably distributed favoring better-off (concentration index = 0.29) and educated women (slope index = 0.47), respectively. Women with mobile phone had significantly higher odds of attending four or more antenatal care visits [adjusted odds ratio (AOR) = 1.42, confidence interval (CI): 95% 1.16, 1.72] and ensuring full immunization in children aged 12–23 months [AOR = 1.61, CI: 95% 1.02, 2.55]. Differences in mobile phone ownership accounted 58% of the observed disparity in antenatal care utilization. These findings underscore that while there has been a substantial increase in mobile phone ownership, it remains unevenly distributed in Rwanda. Addressing these is critical to increasing coverage and equitable access to reproductive and maternal health services in Rwanda.

## INTRODUCTION

Rwanda has made extraordinary strides in achieving the millennium development goals (MDGs) of reducing maternal (750 to 210 per 100 000 live births) and child mortalities (341 to 39 per 100 000 live births) [1]. The country has also the lowest infant mortality rate (28.2 deaths per 1000 live births) in East Africa [2]. Rwanda is committed to attain the maternal and child health–related sustainable development goals (SDGs). Harnessing digital health technologies, is an imperative strategy to ensure optimal access to quality maternal and child health services thereby achieve the SDGs [3, 4]. The rapid adoption of mobile phones in Africa has facilitated the development of digital health tools as a way to improve access to health care services [5]. Yet, many people in the region are facing several real-life barriers to access digital health interventions with overt urban–rural and gender divides, addressing these barriers is essential to improve maternal and child health services and reduce related mortality [6]. Literatures have shown that ownership of mobile phones by women is more likely to impact utilization of reproductive, maternal and child health services [7, 8] and influence success of digital interventions [9].

Rwanda launched the national digital health strategy in 2006 envisioning effective and efficient delivery of healthcare services [10]. The country has made numerous advances in the past two decades to strengthen the use of technology in the health sector [7]. Notwithstanding, Rwanda continues to face inequities in access to reproductive, maternal and child health services, as do most sub-Saharan Africa countries [11]. The maternal mortality has also stagnated from 2015 to 2020, i.e. 210 to 203 deaths per 100 000 live births, respectively. Given the wide acceptance and communal use of mobile phones, they have the capacity to decrease maternal and child mortality through access to health information, timely appointment reminders, improved communication with health providers, and support for informed decision making during pregnancy and child birth [12, 13]. While several studies have evaluated the role of mobile health (mhealth) interventions on reproductive, maternal and child health services [14, 15], there are limited evidences showing links between mobile phones ownership with access to reproductive, maternal and child health services in Rwanda. Generating evidence through this study will provide critical insights to strengthen the current strategies in Rwanda in integrating digital health technologies with reproductive, maternal and child health service delivery and also inform future research initiatives aimed at improving the respective services.

Therefore, this study aimed to examine the trends and socio-economic equity in mobile phone ownership, and whether it contributed to reproductive, maternal and child health service utilization in Rwanda.

## METHODS

### Study design and setting

This study was a secondary analysis of Rwanda demographic and health surveys (DHSs). Rwanda is a low income country in the central eastern Africa with a population size of about 13.7 million [16]. The country is structured into five administrative regions named Kigali, South, West, North and East Regions. The Rwanda health system is organized as a three-level pyramid consisting of the central, intermediate and peripheral levels [17]. Our analyses were based on data from three consecutive Rwanda DHSs of 2010, 2014/15 and 2019/20. The Rwanda DHSs have been conducted every five years to provide updates on the population health and demographics characteristics, and are conducted by the National Institute of Statistics of Rwanda in collaboration with the Ministry of Health and other international organizations [18–20].

### Population and sampling

A two-stage cluster sampling was employed to select study participants in all the three surveys. The first stage involved selecting clusters made up of delineated enumeration areas (EAs). The second stage involved systematic sampling of households. Accordingly, a total of 492 clusters were included in 2010 and 2014/15, while 500 clusters were considered for 2019/20 Rwanda DHS [18–20]. For our analyses, we used the household mobile phone ownership data from 12 792 households from Rwanda DHS 2010 and 2014/15 and 13 000 households from Rwanda DHS 2019/20. We also used 14 634 reproductive aged (15–49 years) women from the latest survey to assess women’s mobile phone ownership. Maternal healthcare utilizations were estimated among women who gave live birth two years prior to each survey. All women of reproductive age and married women were included to assess modern contraceptive uptake and unmet need for family planning, respectively. Moreover, all children aged 12–23 months were included to analyze the coverage of immunization services [18–20].

### Variables

The exposure variable for this study was access to mobile phone. Phone ownership across the three surveys was assessed using the household mobile phone ownership variable stored in the household records (HR) of the three Rwanda DHSs. Women mobile phone ownership was extracted from the latest survey women individual record (IR), and hence it was the only dataset available comprising the variable. Moreover, our analyses were controlled for individual and community level variables included after reviewing literatures [21–26]. Media exposure was assessed by combining whether women had any of these media options, radio, TV, newspaper and magazine. Wealth index was constructed using the selected household asset and analyzed using principal component analysis. Asset scores were categorize into quintiles as poorest, poorer, middle, richer and richest. The community-level variables included were community’s education status, media exposure, mobile phone ownership and wealth status.

Reproductive, maternal and child healthcare utilizations were the outcomes of interest, defined taking into account the World Health Organization (WHO) tracer indicators for the respective services [27]. Antenatal care (ANC) utilization was measured as proportion of women who received four or more ANC visits for the most recent pregnancy. Early initiation of ANC was defined as women who started the first ANC visit during the first trimester of the pregnancy or in 12 weeks of gestation. Skilled Birth Attendance (SBA) was defined as births delivered by a skilled provider, a doctor, nurse, auxiliary midwife, community health worker or mother and child-community health worker. We estimated facility delivery as percentage of women who gave birth at public or private health facility. Postnatal care (PNC) utilization was calculated as proportion of mother who had first postnatal check for the most recent live birth within 24 hours after delivery.

The modern contraceptive use was estimated if a woman or her partner used any type of modern contraceptive methods, i.e. pills, IUD, injections, implants/Norplant, male and female sterilization, male and female condoms, lactational amenorrhea (lam) and emergency contraception. Unmet needs for family planning was defined as proportion of women who desired to either delay the next pregnancy or limit future pregnancies, but were not using any method of contraception. Children aged 12–23 months were considered fully immunized when they received BCG, three doses of polio vaccine, three doses of pentavalent and one dose of measles vaccine.

### Data management and analyses

The individual women record, kid’s record and HR data set was cleaned, categorized and checked for completeness before going to the analyses stage. Descriptive statistics were carried out and summarized using tables, texts and graphs. Each outcome variable was dichotomized. The household mobile phone ownership was merged with mothers’ and children’s data sets for each survey. The household and individual merged data sets of each survey (2010, 2014/15 and 2019/20) were appended to create one large data sets for the trend analysis. Analyses were adjusted for sampling weight and cluster variations. Weighting was done using the standard DHS weight variable (v005 for women’s data and, hv005 for household data), divided by 1 000 000. We also examined the changes in access to mobile phone and the coverage of reproductive, maternal and child health services overtime using trend analyses (2010–2019/20).

The socio economic equality in mobile phone ownership was assessed using both absolute and relative equity measures. Household wealth and women’s education were used as stratifiers for the social equity analysis. The positive and negative equity analysis indexes [slope of index (SI) and concentration index] suggest inequities favoring socially advantaged and disadvantaged groups, respectively. Equiplots were used to graphically illustrate inequities in mobile phone ownership. Given the hierarchical data structures with individuals nested under geographical clusters (primary sampling units), a multilevel logistic regression was fitted to identify the association of reproductive, maternal and child health service utilization with women’s mobile phone ownership using the latest data, 2019/20 Rwanda DHS. Four models were fitted for each outcome and the null model, which contained no exposure variable, was used to assess the variability of the study outcomes. Individual- and community-level variables were included in the first (Model 1) and second (Model 2) multilevel models, respectively. Individual and community-level variables were fitted simultaneously with the occurrence of the outcomes in the third model (Model 3). The interclass correlation coefficients (ICCs) were used to compare models; hence the model that well explained the variation was chosen as the best-fitted model. Finally, variables with a *P*-value of ≤0.25 were selected as candidates for the final model. Crude odds ratio (OR) and adjusted odds ratio (AOR) with a 95% confidence interval (CI) were calculated to measure the strength of association between the outcomes and independent variables.

Decomposition analysis was employed to identify the contributing factors to the disparity in healthcare utilizations across women with and without phone ownership for the outcomes that showed significant association in the multilevel analysis. The decomposition model additively explains the difference in health care utilizations among women with and without mobile phone ownerships by ‘Endowments’ (i.e. the difference in health care utilizations is due to the difference in the selected variables of women between mobile phone owners and non-owners, which is explained) and ‘Coefficients’ (i.e. difference in health care utilizations is due to the effects of those selected variable, that are unexplained). Age, educational status, residence, wealth index and parity were used to explain the disparities. The results were displayed in bar graphs showing percentages of endowment and coefficient. Statistical significance of association in the decomposition analysis and other statistical models were declared at a *P*-value of <0.05.

### Ethics approval

All Rwanda DHSs were ethically approved by the Rwanda National Ethics Committee (RNEC) and the International Coaching Federation Institutional Review Board. Written informed consent was obtained from all women before data collection, and all the data were anonymized from individual’s names, addresses and other identifiers. Approval for the use of data was sought and received from the DHS program https://www.dhsprogram.com/. We confirm that the study was conducted according to the Declaration of Helsinki.

## RESULTS

### Characteristics of the study participants

A total of 13 671(Rwanda DHS 2010), 13 497(Rwanda DHS 2014/15) and 14 634(Rwanda DHS 2019/20) women of reproductive age were included in the analysis. Of these 3101, 3136 and 3054 gave birth in two years before each survey, respectively. Nearly one-third of women were 35 years or older in three surveys. Proportion of women with secondary or higher education increased from 16% in 2010 to 32% in 2019/20. Two- thirds of the participants reported males as a household head in all surveys. More than three-fourths resided in the rural areas (Table 1).

**Table 1:** Socio-demographic characteristics of the study participants

Characteristics	Frequency (%)^*^ 2010 *n* = 13 671	Frequency (%)^*^ 2014/15 *n* = 13 497	Frequency (%)^*^ 2019/20 *n* = 14 634
Women’s age			
15–19	2963 (22)	2779 (21)	3308 (22)
20–24	2692 (20)	2473 (18)	2424 (16)
25–29	2495 (18)	2319 (17)	2047 (14)
30–34	1822 (13)	2155 (16)	2095 (14)
≥35	3699 (27)	3771 (28)	4760 (33)
Sex of household head			
Male	9088 (66)	9249 (69)	10 045 (69)
Female	4583 (34)	4248 (31)	4589 (31)
Marital status			
Not in union^**^	6837 (50)	6607 (48)	7344 (49)
married/living with partner	6834 (50)	6890 (52)	7290 (51)
Highest level of women’s education			
No education	2061 (16)	1665 (12)	1352 (9)
Primary	9277 (68)	8509 (65)	8500 (58)
Secondary or higher	2333 (16)	3388 (23)	4782 (33)
Occupational status			
Not working	2295 (16)	2020 (15)	3955 (27)
Working	11 376 (84)	11 477 (85)	10 679 (73)
Wealth index			
Poorest	2569 (19)	2523 (18)	2844 (19)
poorer	2603 (19)	2516 (20)	2707 (19)
Middle	2663 (20)	2461 (19)	2709 (19)
Richer	2621 (20)	2523 (20)	2884 (20)
Richest	3215 (22)	3474 (23)	3490 (23)
Media exposure			
Yes	922 (7)	1653 (13)	2568 (17)
No	12 749 (93)	11 784 (87)	12 066 (83)
Residence			
Urban	2367 (15)	3427 (19)	3551 (20)
Rural	11 304 (85)	10 070 (81)	11 083 (80)
Region			
Kigali city	1890 (12)	1876 (14)	1921 (15)
South	3340 (24)	3435 (24)	3482 (21)
West	3138 (23)	3.060 (22)	3312 (22)
North	2199 (17)	2170 (15)	2294 (15)
East	3104 (24)	2956 (25)	3625 (27)

^*^ *Unweighted frequency and weighted percentages*. ^**^Single, widowed, separated, divorced.

### Mobile phone ownership

Household mobile phone ownership in Rwanda increased from 40% (95%CI: 39–41) in 2010 to 71% (95%CI: 70 to 72) in 2019/20. While, only 48% (95% CI: 47%, 49%) of women had mobile phone in 2019/20.

### Socio-economic equity in mobile phone ownership

**Figure 1:**
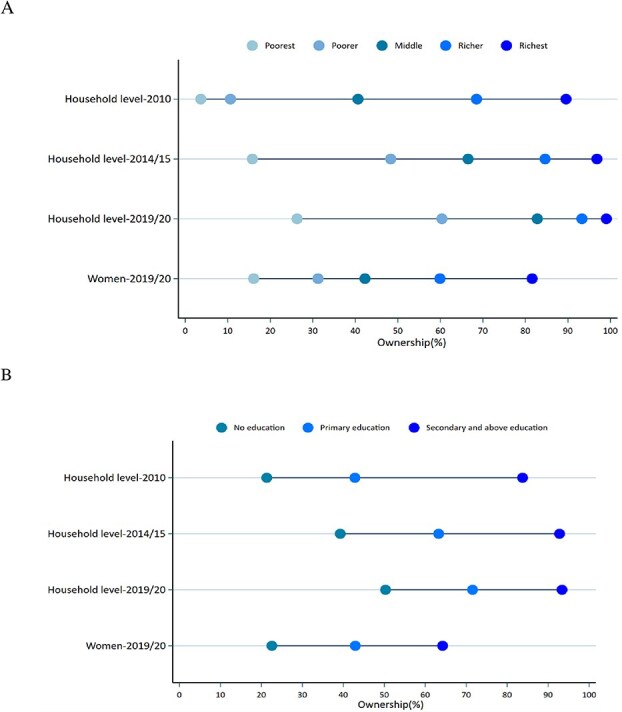
Equiplots for mobile phone ownerships based on wealth (a) and education (b) groups 2010–2019/20.

Findings showed wealth- and education-based inequities in mobile phone ownership from 2010 to 2019/20, but the observed disparities decreased over time (Fig. 1). Mobile phone ownership tended to be higher among the better-off and educated households. The absolute gaps in household mobile phone ownership decreased from 86% to 73% between poorest and richest wealth quintiles in 2010 and 2019/20, respectively (Table 2). Similarly, the gaps in household mobile phone ownership among study participants with no schooling and secondary or higher education decreased from 62% to 44% in the same periods (Table 2). Similar pro-rich and pro-educated social inequities in women’s mobile phone ownership were reported with 66% difference between women in the poorest and richest category, and 42% difference among women with no education and those with secondary or higher education level.

**Table 2:** Relative and absolute inequities in ownership of mobile phones across years in Rwanda

Dimensions	Category	Household mobile phone ownership			Women’s mobile phone ownership
		2010	2014/15	2019/20	2019/20
Wealth index	Slope index/SI				
	Coefficient (SE)	0.92 (0.003)	0.86 (0.005)	0.83 (0.006)	0.73 (0.008)
	*P*-value	< 0.001	< 0.001	< 0.001	< 0.001
	Difference (Q5-Q1; % points)	86	81	73	66
	Concentration index				
	Coefficient (SE)	0.46 (0.004)	0.28 (0.280)	0.22 (0.003)	0.29 (0.004)
	*P*-value	< 0.001	< 0.001	< 0.001	< 0.001
	Ratio (Q5: Q1)	25	6	4	5
Education	Slope index/SI				
	Coefficient (SE)	0.63 (0.012)	0.59 (0.012)	0.50 (0.012)	0.47 (0.013)
	*P*-value	< 0.001	< 0.001	< 0.001	< 0.001

^*^SE: Standard error.

The SI analyses showed pro-rich social inequity in household’s mobile phone ownership which decreased over time, in 2010 (SI = 0.92, *P*-value<0.001), 2014/15 (SI = 0.86, *P*-value<0.001) and 2019/20 (SI = 0.73, *P*-value<0.001). The relative equity analyses also showed that mobile phone ownership was disproportionately higher among better-off households, with respective concentration index of 0.46 in 2010, 0.28 in 2014/2015, 0.22 in 2019/2020 (Table 2). Results also indicated that mobile phone ownership appeared to be largely concentrated among the educated households across all survey years with SI = 0.63 (*P*-value<0.001) in 2010, 0.59 (*P*-value<0.001) in 2014/15 and 050 (*P*-value<0.001) in 2019/20. Furthermore, our findings revealed inequities in mobile phone ownership favoring the better-off (concentration index = 0.29, *P*-value<0.001) and pro-educated women (SI of 0.47 at a *P*-value of<0.001), respectively (Table 2).

**Figure 2:**
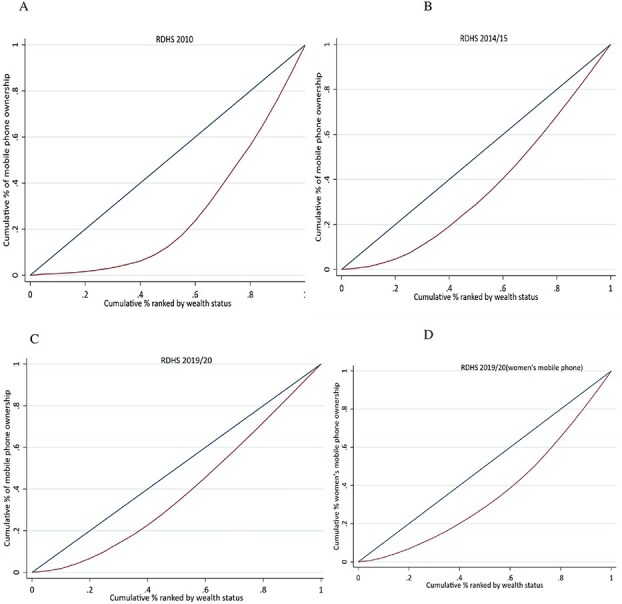
Wealth based inequity of household (a, b, c) and women’s (d) mobile phone ownership from 2010 to 2019/20.

All the concentration curves lied below the line of equity, indicating that the mobile ownership was inequitably concentrated among better-off women than their poorest counterparts. Compared to the previous years, the concentration curves were relatively closer to the equity line in 2019/20, indicating that socio-economic inequities were decreasing overtime (Fig. 2).

### Trends in reproductive, maternal and child healthcare utilization with mobile phone ownership

Our trend analysis illustrated significant (*P*-values of <0.001) increment in maternal healthcare utilization as the household mobile phone ownership increased over the study periods, 2010–2019/20. The largest increment was seen in early PNC utilization with 52 point change, 15% (95%CI: 14, 16.) in 2010 to 67% (95% CI: 65, 68) in 2019/20. The least increment was observed in four or more ANC utilization with only 12 percentage point change between 2010 and 2019/20. Slight increase in modern contraceptive use and decrease in unmet need for family planning was illustrated in the study periods. However, there was no statistically significant trend in improvements in modern contraceptive use (*P*-value = 0.15) and decrease in unmet need for family planning (*P*-value = 0.58) in the survey periods. The coverage of child full immunization significantly increased with improvements in household mobile phone ownership over time, *P*-value of <0.001.

### Association of women’s mobile phone ownership with reproductive maternal and child health care utilization

The adjusted multivariable multilevel logistic regression analysis showed that women’s mobile phone ownership was significantly associated with ANC utilization. The odds of early initiation (AOR = 1.37, 95% CI: 1.11, 1.67) and four or more ANC visits (AOR = 1.42, 95% CI: 1.16, 1.72) were significantly higher among women who had mobile phone compared to those who did not have. The crude analysis revealed statistically significant association of women’s mobile phone ownership with skilled or facility delivery and PNC visit. However, the observed association was not apparent when analysis was controlled for women and household background characteristics (Table 3). However, women’s mobile phone ownership was not significantly associated with modern contraceptive use and unmet need for family planning. Children of women who had mobile phone had significantly higher odds of receiving all basic immunization (AOR = 1.61, 95% 1.02, 2.55) (Table 3).

**Table 3:** Association between mobile phone ownership and healthcare utilization.

Outcomes	Mobile phone owners	Non mobile phone owners	Crude OR 95% CI	Adjusted OR 95% CI
Antenatal care^#^				
ANC ≥4	763 (56%)	739 (41%)	1.86 (1.59, 2.19)	1.42^**^ (1.16, 1.72)
ANC <4	599 (44%)	1053 (59%)	1.00	1.00
Early ANC initiation^#^				
Yes	873 (65%)	914 (53%)	1.65 (1.41, 1.93)	1.37^*^ (1.11, 1.67)
No	480 (35%)	815 (47%)	1.00	1.00
Skilled birth attendance^#^				
Yes	1330 (98%)	1672 (93%)	2.84 (1.85, 4.36)	1.04 (0.61 1.78)
No	32 (2%)	121 (7%)	1.00	1.00
Facility delivery^#^				
Yes	1319 (97%)	1656 (92%)	2.35 (1.60, 3.43)	0.85 (0.53, 1.38)
No	43 (3%)	138 (8%)	1.00	1.00
PNC utilization^#^				
Yes	968 (71%)	1133 (63%)	1.50 (1.26, 1.79)	1.17 (0.94, 1.45)
No	394 (29%)	660 (37%)	1.00	1.00
Modern contraceptive use^#^				
Yes	2407 (37%)	2656 (38%)	1.03 (0.93, 1.08)	—
No	4048 (63%)	4324 (62%)	1.00	
Unmet need of family planning^#^				
Yes	484 (13.71%)	525 (13.57%)	1.00 (0.87, 1.55)	—
No	3046 (86.29%)	3346 (86.43%)	1.00	
Full immunization^#^				
Yes	620 (87%)	757 (82%)	1.77 (1.22, 2.58)	1.61^*^ (1.02, 2.55)
No	91 (13%)	164 (18%)	1.00	1.00

^**^ *P*-value < 0.001.

^*^ *P*-value < 0.05.

^#^Analyses was adjusted for range of individual and community level factors.

### Factors associated with mobile phone ownership disparity

**Figure 3:**
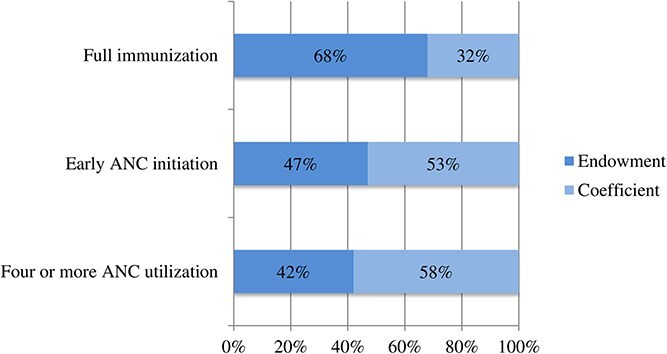
Explained differences between women with and without mobile phones across healthcare utilizations.

Using the outcomes which had a significant association with mobile phone ownership, we further decomposed the contribution of determinants for health service utilization disparity across mobile phone ownership. The statistical estimates for contribution were shown in endowment and coefficients suggesting differences between women with and without phone access in the magnitude of their background characteristics like wealth, education, residence, age and parity and differences in their ownership to phone, respectively. Accordingly, ~42% and 47% of the gap was explained by background characteristics, while 53% and 58% were due to the gap in women’s mobile ownership for early initiation and four or more ANC utilization, respectively. Nevertheless, both the endowment and coefficient components were insignificant in explaining gaps for child full immunization coverage (Fig. 3).

## DISCUSSION

Household’s access to mobile phone significantly increased in the last decade with overt social inequities favoring better-off and educated families. Women’s access to mobile phone was associated with increased utilization of ANC and child immunization services. Disparities in access to mobile phone explained most of the observed inequities in maternal health care utilization.

We used three nationally representative datasets and applied varied equity analysis techniques which provided a deeper and comprehensive insight on disparities in women’s access to mobile phone and its contribution to utilization of health care services. To enhance memory, we assessed maternal health care utilization among those who gave birth in the last two years prior to the study. Child immunization was also estimated mainly using immunization card review and field workers also considered different interview techniques (such as reminding about route of administration) to enhance their memory. Despite this, recall bias could be a possible limitation in estimating the coverage of maternal health and child immunization services. All analyses were based on cross-sectional data, which limits causal inferences. Data on household or women’s mobile phone ownership might have a limitation of showing whether or to what extent they are using the devices to receive health information. Moreover, the data does not typically specify between ownership of a smartphone or feature phone. Additionally, ownership as measured in DHS may not reflect exclusive or consistent access by women, especially in rural and low-income households, where phones might be shared.

Our findings showed that two-thirds of households had mobile phone in Rwanda. There was marked improvement in household’s access to mobile phone in the last one decade, 2010 to 2019/20. This was in line with the reported average for mobile phone ownership for sub-Saharan Africa (63%) [28]. The observed increase in mobile phone access could be attributed to Rwanda’s remarkable investment in telecommunication infrastructure [29]. Despite the reported changes in mobile phone ownership, one-third of households did not have access to mobile phone, suggesting that this group of people might have decreased access to digital health information. This may also worsen the existing social inequities in access to health care services [30]. Results of this study showed significant gender-based and socio-economic inequity in access to mobile phone among Rwandan population. Mobile phone ownership was significantly lower among women, poor and less educated families. A report in low- and middle-income country illustrated gender-based inequities disfavoring women in access to mobile phone [7]. Pro-rich and educated inequity in mobile phone ownership was also shown in other low- and middle-income countries [7, 31]. Deep-rooted cultural norms disfavoring women in Africa might explain the observed gender-based inequities in mobile phone ownership [32]. Disparities in digital health literacy and variations in purchasing power underlie the socio-economic inequity in access to mobile phone in socially disadvantaged Rwandan population [31]. This suggests that women, poor and less educated Rwandans have lesser access to digital health information. Strengthening Rwanda effort in addressing gender-based and social inequities is critical to ensure optimal awareness and utilization of essential health care services in socially disadvantaged families [33, 34].

Findings from the trend analysis showed a significant association between the increasing patterns in household mobile phone ownership and utilization of maternal and child health services. This highlights the transformative potential of mobile technology in improving healthcare access through addressing economic, geographic and infrastructural barriers [5]. Our results revealed higher likelihood of early initiation and coverage of four or more ANC visits. The positive effect of mobile phone ownership on number of ANC attendance was also shown by the previous studies in other low- and middle-income countries [35, 36]. Women’s use of mobile phones opens up opportunities for them to receive health information on ANC services and remainders for the respective follow-ups [37]. Delivery of mhealth intervention through SMS was found to improve maternal health care services, including ANC [38]. ANC is one of the most critical windows of opportunity to promote health and well-being and thereby enhancing the survival of mothers and children [39]. The implementation of mhealth interventions is mainly dependent on infrastructure availability, such as mobile phones and internet connectivity. Countries investment on digital health infrastructures is a key to scale-up and ensure accessibility of mhealth innovations [40]. Since 2009, the government of Rwanda has endorsed different digital health strategies [41]. Although ANC coverage stands at 98% in Rwanda DHS 2019/20, four or more ANC utilization was only 48%, which further is supported by a study done in Africa that shows Rwanda to have the lowest optimal ANC utilization [42]. Rwanda has been at the forefront of leveraging digital health to improve maternal and child health outcomes, with initiatives like RapidSMS and the national strategies supporting real-time monitoring and communication for maternal and child health services [10, 14]. However, gaps remain in the effective use of these digital tools. Further intensifying Rwanda’s effort in expanding digital health infrastructures and access to mobile phone and internet connectivity will have great potential to improve the reported inadequate coverage for optimal four or more ANC follow-ups. This could also be an added benefit to address inadequate access to health care services related to shortage of health care professionals in the country [43]. Our further analysis revealed that variations in in women’s access to mobile phone explained most of the disparities in ANC utilization [44]. This might further exacerbate the existing disparities in health care access. Giving special priority to disadvantaged population groups need to be an important component of strategies aiming to ensure optimal access to digital health interventions and essential health care services [45].

The study also highlights a significant link between women’s mobile phone ownership and utilization of child immunization service. Previous study has highlighted the importance of mobile phone ownership in increasing women’s decision-making power and facilitating the exchange of information which could directly improve parents’ health literacy thereby inform how to care for their children [46]. Mobile text messages and phone call remainders were found increasing uptake of child vaccination services in Africa [47]. Rwanda has been an exemplar African country in reaching near universal childhood immunization coverage, and implementation of digital health innovations partly under lied this success [48]. Rwanda achievement in supporting child immunization service delivery with digital health technologies underscore critical lesson to enhance the low coverage of child immunization in sub-Saharan Africa through improving parents’ vaccination literacy, readiness to use the service and addressing their forgetfulness for schedules [49].

## CONCLUSIONS

Household access to mobile phone significantly increased over time in Rwanda. There were significant relationships in improvements in access to mobile phone with an increase in the coverage of maternal and child health services. Access to mobile phone was inequitably distributed favoring the pro-rich and educated women or families. Only half of Rwandan women had access to mobile phone. The coverage of four or more ANC and child immunization was significantly higher among women who had access to mobile phone. Differences in phone ownership under lied most of the disparities in ANC utilizations. These findings suggest the importance of further strengthening implementation of digital interventions to optimize access to mobile phone in Rwanda. Improving mobile phone affordability and access for underserved groups particularly poor and less educated women and families is crucial for expanding coverage of reproductive, maternal and child health services. Governments and development partners should also leverage market mechanisms to stimulate private sector solutions while deploying targeted public subsidies to address gaps where the market fails, ensuring equitable access to mobile technology for those most in need. Mitigating gender-related barriers could also help to further improve women’s access to mobile phone. In a nutshell, intensifying the implementation of digital health interventions and its integration with the primary health care system will have pivotal importance to accelerate Rwanda’s progress toward attaining maternal and child health–related SDGs. Future research should explore how mobile phone access translates into the actual use, considering quality, frequency and autonomy of use among women particularly in disadvantaged setting. Studies could also assess whether digital interventions can reduce the observed disparities in ANC and immunization coverage, and explore causal pathways through longitudinal or implementation research.

## Data Availability

Data used in this study are publically available through the Demographic and Health Survey Programme at https://www.dhsprogram.com/.

## References

[1] Gurusamy PSR, Janagaraj PD. A success story: the burden of maternal, neonatal and childhood mortality in Rwanda—critical appraisal of interventions and recommendations for the future. *Afr J Reprod Health* 2018;22:9–16. 10.29063/ajrh2018/v22i2.1 30052329

[2] Kitua A . Health research agenda for East Africa in the new millennium: looking ahead. *Tanzania J Health Res* 2007;9:147–53. 10.4314/thrb.v9i3.14319 18087890

[3] Asi YM, Williams C. The role of digital health in making progress toward sustainable development goal (SDG) 3 in conflict-affected populations. *Int J Med Inform* 2018;114:114–20. 10.1016/j.ijmedinf.2017.11.003 29126701

[4] Till S, Mkhize M, Farao J et al. Digital health technologies for maternal and child health in Africa and other low-and middle-income countries: cross-disciplinary scoping review with stakeholder consultation. *J Med Internet Res* 2023;25:e42161. 10.2196/42161 37027199 PMC10131761

[5] McCool J, Dobson R, Whittaker R et al. Mobile health (mHealth) in low-and middle-income countries. *Annu Rev Public Health* 2022;43:525–39. 10.1146/annurev-publhealth-052620-093850 34648368

[6] Holst C, Sukums F, Radovanovic D et al. Sub-Saharan Africa—the new breeding ground for global digital health. *Lancet Dig Health* 2020;2:e160–2. 10.1016/S2589-7500(20)30027-3 33328076

[7] LeFevre AE, Shah N, Bashingwa JJH et al. Does women’s mobile phone ownership matter for health? Evidence from 15 countries. *BMJ Glob Health* 2020;5:e002524. 10.1136/bmjgh-2020-002524 PMC7245424 32424014

[8] Mohan D, Bashingwa JJH, Tiffin N et al. Does having a mobile phone matter? Linking phone access among women to health in India: an exploratory analysis of the national family health survey. *PLoS One* 2020;15:e0236078. 10.1371/journal.pone.0236078 32687527 PMC7371204

[9] Choudhury A, Nimbarte A. Mobile health interventions to address maternal health: ideas, concepts, and interventions. *Front Digit Health* 2024;6:1378416. 10.3389/fdgth.2024.1378416 38486918 PMC10937536

[10] Rwanda Digital Health Strategic Plan 2018–2023. https://extranet.who.int/countryplanningcycles/sites/default/files/public_file_rep/RWA_Rwanda_Digital-Health-Strategy_2018-2023.Pdf.

[11] Rulisa S, Ntihinyurwa P, Ntirushwa D et al. Causes of maternal mortality in Rwanda, 2017–2019. *Obstet Gynecol* 2021;138:552–6. 10.1097/AOG.0000000000004534 34623066

[12] O’Brien N, Li E, Chaibva CN et al. Strengths, weaknesses, opportunities, and threats analysis of the use of digital health technologies in primary health care in the sub-Saharan African region: qualitative study. *J Med Internet Res* 2023;25:e45224. 10.2196/45224 37676721 PMC10514769

[13] Mildon A, Sellen D. Use of mobile phones for behavior change communication to improve maternal, newborn and child health: a scoping review. *J Glob Health* 2019;9:020425. 10.7189/jogh.09.020425 31893032 PMC6925966

[14] Ngabo F, Nguimfack J, Nwaigwe F et al. Designing and implementing an innovative SMS-based alert system (RapidSMS-MCH) to monitor pregnancy and reduce maternal and child deaths in Rwanda. *Pan Afr Med J* 2012;13:31. 23330022 PMC3542808

[15] Ruton H, Musabyimana A, Gaju E et al. The impact of an mHealth monitoring system on health care utilization by mothers and children: an evaluation using routine health information in Rwanda. *Health Policy Plan* 2018;33:920–7. 10.1093/heapol/czy066 30169638 PMC6172419

[16] characteristics, P.s.a.P. https://www.statistics.gov.rw/statistical-publications/subject/population-size-and-population-characteristics.

[17] MOH-Rwanda . Health Service Packages for Public Health Facilities [cited 28 June 2024]; https://www.moh.gov.rw/.

[18] Lee RY, Landau AY, Heider PM et al. Estimating the prevalence of child abuse and neglect among adolescents in primary care through diagnoses codes and free-text EHR clinical notes. *J Pediatr Health Care* 2025;39:189–97. 10.1016/j.pedhc.2024.10.016 39580745 PMC11807751

[19] Chauhadry IA, Soofi SB, Sajid M et al. Bridging the vaccination equity gap: a community-driven approach to reduce vaccine inequities in polio high-risk areas of Pakistan. *Vaccines* 2024;12:1340. 10.3390/vaccines12121340 39772002 PMC11680313

[20] Levin A, Fisseha T, Reynolds HW et al. Scoping review of current costing literature on interventions to reach zero-dose children in low- and middle-income countries. *Vaccines* 2024;12:1431. 10.3390/vaccines12121431 39772091 PMC11728644

[21] Tessema ZT, Teshale AB, Tesema GA et al. Determinants of completing recommended antenatal care utilization in sub-Saharan from 2006 to 2018: evidence from 36 countries using demographic and health surveys. *BMC Pregnancy Childbirth* 2021;21:1–12. 10.1186/s12884-021-03669-w 33676440 PMC7937261

[22] Woldegiorgis MA, Hiller J, Mekonnen W et al. Determinants of antenatal care and skilled birth attendance in sub-Saharan Africa: a multilevel analysis. *Health Serv Res* 2019;54:1110–8. 10.1111/1475-6773.13163 31090931 PMC6736910

[23] Negash WD, Eshetu HB, Asmamaw DB. Predictors of modern contraceptive use among reproductive age women in high fertility countries in sub-Saharan Africa: evidence from demographic and health surveys. *BMC Womens Health* 2022;22:520. 10.1186/s12905-022-02121-1 36514075 PMC9746200

[24] Teshale AB . Factors associated with unmet need for family planning in sub-Saharan Africa: a multilevel multinomial logistic regression analysis. *PLoS One* 2022;17:e0263885. 10.1371/journal.pone.0263885 35143584 PMC8830726

[25] Fenta SM, Biresaw HB, Fentaw KD et al. Determinants of full childhood immunization among children aged 12–23 months in sub-Saharan Africa: a multilevel analysis using demographic and health survey data. *Trop Medicine Health* 2021;49:1–12. 10.1186/s41182-021-00319-x PMC8017626 33795028

[26] Appiah F, Salihu T, Fenteng JOD et al. Factors influencing early postnatal care utilisation among women: evidence from the 2014 Ghana demographic and health survey. *PLoS One* 2021;16:e0249480. 10.1371/journal.pone.0249480 33798224 PMC8018634

[27] Wehrmeister FC, Barros AJD, Hosseinpoor AR et al. Measuring universal health coverage in reproductive, maternal, newborn and child health: an update of the composite coverage index. *PLoS One* 2020;15:e0232350. 10.1371/journal.pone.0232350 32348356 PMC7190152

[28] Integration of Stepped Care for Perinatal Mood and Anxiety Disorders Among Women Attending MCH Clinics A1—Anonymous *.* clinicaltrials.gov, 2024.

[29] Caldarola B, Grazzi M, Occelli M et al. Mobile internet, skills and structural transformation in Rwanda. *Res Policy* 2023;52:104871. 10.1016/j.respol.2023.104871

[30] Blake A, Hazel A, Jakurama J et al. Disparities in mobile phone ownership reflect inequities in access to healthcare. *PLOS Digit Health* 2023;2:e0000270. 10.1371/journal.pdig.0000270 37410708 PMC10325035

[31] Blumenstock, J. and N. Eagle. *Mobile divides: Gender, socioeconomic status, and mobile phone use in Rwanda*. in Proceedings of the 4th ACM/IEEE international conference on information and communication technologies and development. 2010.

[32] Well-Child Care Clinical Practice Redesign: A Parent Coach-Led Model of Care for Young Children A1—Anonymous *.* clinicaltrials.gov, 2018.

[33] Rotondi V, Kashyap R, Pesando LM et al. Leveraging mobile phones to attain sustainable development. *Proc Natl Acad Sci* 2020;117:13413–20. 10.1073/pnas.1909326117 32482867 PMC7306795

[34] Musizvingoza R, Handforth C. The Digital Gender Gap in HealthCare: Progress, Challenges, and Policy Implications, 2021.

[35] Lund S, Nielsen BB, Hemed M et al. Mobile phones improve antenatal care attendance in Zanzibar: a cluster randomized controlled trial. *BMC Pregnancy Childbirth* 2014;14:1–10. 10.1186/1471-2393-14-29 24438517 PMC3898378

[36] Kibria GMA, Hashan MR, Hanif AAM et al. Mobile phone use for pregnancy-related healthcare utilization and its association with optimum antenatal care and hospital delivery in Bangladesh. *PLOS Glob Public Health* 2023;3:e0001762. 10.1371/journal.pgph.0001762 37022996 PMC10079009

[37] Van Den Heuvel , Groenhof TK, Veerbeek JH et al. eHealth as the next-generation perinatal care: an overview of the literature. *J Med Internet Res* 2018;20:e202. 10.2196/jmir.9262 29871855 PMC6008510

[38] Feroz A, Perveen S, Aftab W. Role of mHealth applications for improving antenatal and postnatal care in low and middle income countries: a systematic review. *BMC Health Serv Res* 2017;17:1–11. 10.1186/s12913-017-2664-7 29115992 PMC5678803

[39] Kanyangarara M, Munos MK, Walker N. Quality of antenatal care service provision in health facilities across sub-Saharan Africa: evidence from nationally representative health facility assessments. *J Glob Health* 2017;7. 10.7189/jogh.07.021101 PMC5680531 29163936

[40] Erku D, Khatri R, Endalamaw A et al. Digital health interventions to improve access to and quality of primary health care services: a scoping review. *Int J Environ Res Public Health* 2023;20:6854. 10.3390/ijerph20196854 37835125 PMC10572344

[41] Tankala R, Huang L, Hiskens M et al. Neonatal retrievals from a regional centre: outcomes, missed opportunities and barriers to back transfer. *J Paediatr Child Health* 2023;59:680–5. 10.1111/jpc.16370 36799108

[42] Raru TB, Ayana GM, Zakaria HF et al. Association of higher educational attainment on antenatal care utilization among pregnant women in east Africa using demographic and health surveys (DHS) from 2010 to 2018: a multilevel analysis. *Int J Women's Health* 14:67–77. 10.2147/IJWH.S350510 PMC8819274 35140524

[43] Aboye GT, Vande Walle M, Simegn GL et al. Current evidence on the use of mHealth approaches in sub-Saharan Africa: a scoping review. *Health Policy Technol* 2023;12:100806. 10.1016/j.hlpt.2023.100806

[44] Jennings L, Omoni A, Akerele A et al. Disparities in mobile phone access and maternal health service utilization in Nigeria: a population-based survey. *Int J Med Inform* 2015;84:341–8. 10.1016/j.ijmedinf.2015.01.016 25737460

[45] Ryu S . Book review: mHealth: new horizons for health through mobile technologies: based on the findings of the second global survey on eHealth (global observatory for eHealth series, volume 3). *Healthcare Inform Res* 2012;18:231. 10.4258/hir.2012.18.3.231

[46] Flückiger M, Ludwig M. Mobile phone coverage and infant mortality in sub-Saharan Africa. *J Econ Behav Organ* 2023;211:462–85. 10.1016/j.jebo.2023.05.013

[47] Gilano G, Sako S, Molla B et al. The effect of mHealth on childhood vaccination in Africa: a systematic review and meta-analysis. *PLoS One* 2024;19:e0294442. 10.1371/journal.pone.0294442 38381753 PMC10880990

[48] Bao J, McAlister H, Robson J et al. Near universal childhood vaccination rates in Rwanda: how was this achieved and can it be duplicated? *Lancet Glob Health* 2018;6:S47. 10.1016/S2214-109X(18)30176-1

[49] Bangura JB, Xiao S, Qiu D et al. Barriers to childhood immunization in sub-Saharan Africa: a systematic review. *BMC Public Health* 2020;20:1108. 10.1186/s12889-020-09169-4 32664849 PMC7362649

