# Correction to: Access to mobile phone, socio-economic equity and maternal and child healthcare utilization in Rwanda: analysis of demographic and health surveys

**DOI:** 10.1093/oodh/oqaf033

**Published:** 2025-12-02

This is a correction to: Amare Tariku, Betelhem Abebe Andargie, Tesfahun Melese Yilma, Abdulaziz Mohammed, Admas Abera, Diwakar Mohan, Shivani Pandya, Lena Kan, Hinda Ruton, Meredith Kimball, Patricia Mechael, Smisha Agarwal, Binyam Tilahun, Access to mobile phone, socio-economic equity and maternal and child healthcare utilization in Rwanda: analysis of demographic and health surveys, *Oxford Open Digital Health*, Volume 3, 2025, https://doi.org/10.1093/oodh/oqaf018

The following changes have been made to the originally published manuscript.

The name of the fifth author has been emended to read: “Admas Abera”.

In addition to the corrections in axes labelling for years (Figs 1 and 2) and legend emendation (Fig. 3), Figures 1 and 2 are corrected of errors in the originally-plotted graphs, and the data presentation in Figure 3 is reformatted, in order to align all with the descriptions and interpretations already reflected in the manuscript text.

Figure 1 should read:


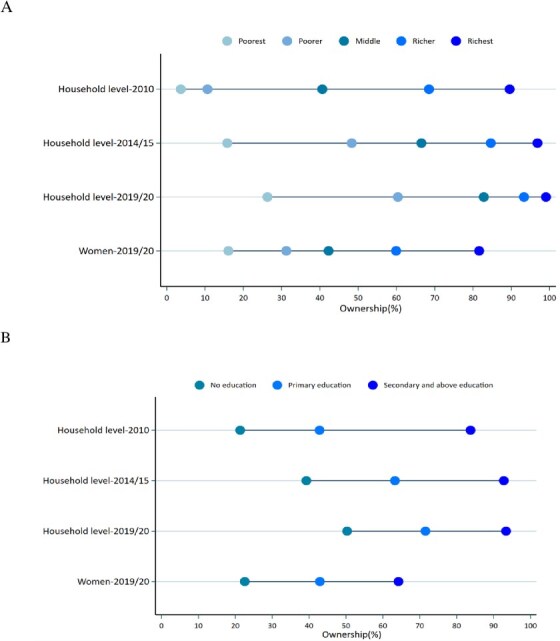


instead of:


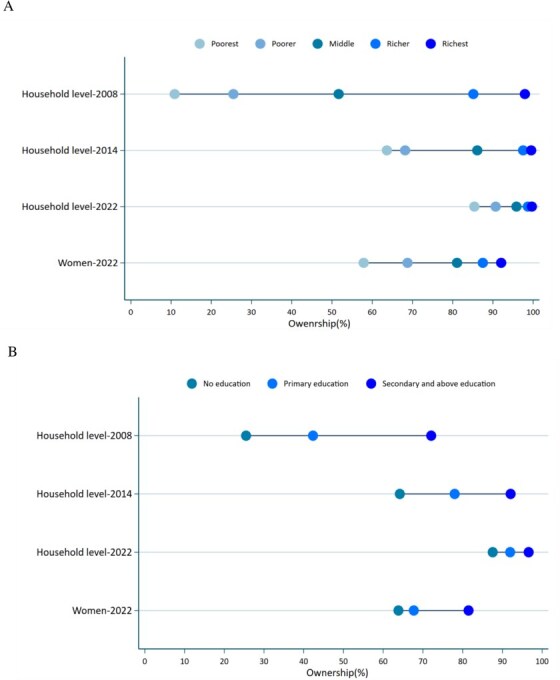


Figure 2 should read:


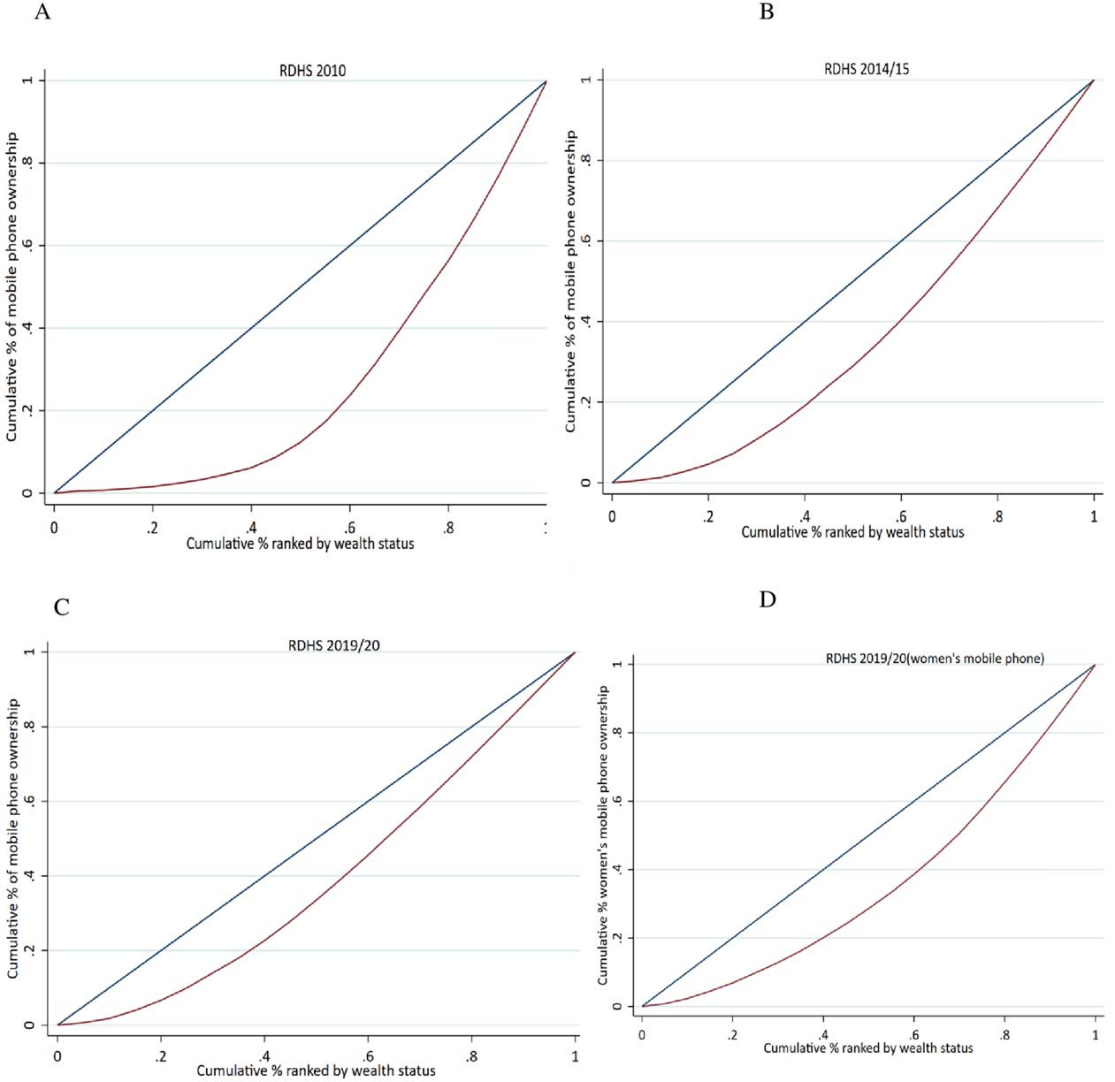


instead of:


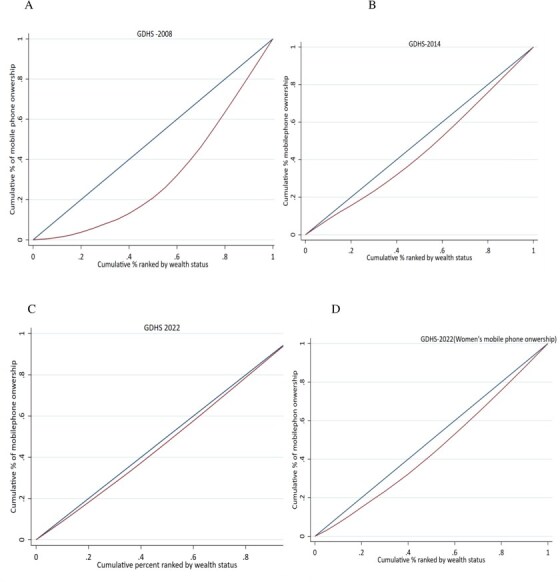


Errors in the legend of Figure 3 is emended to read: “Endowment” and “Coefficient” instead of: “Endwoment” and “Coefficnet”.

Figure 3 should read:


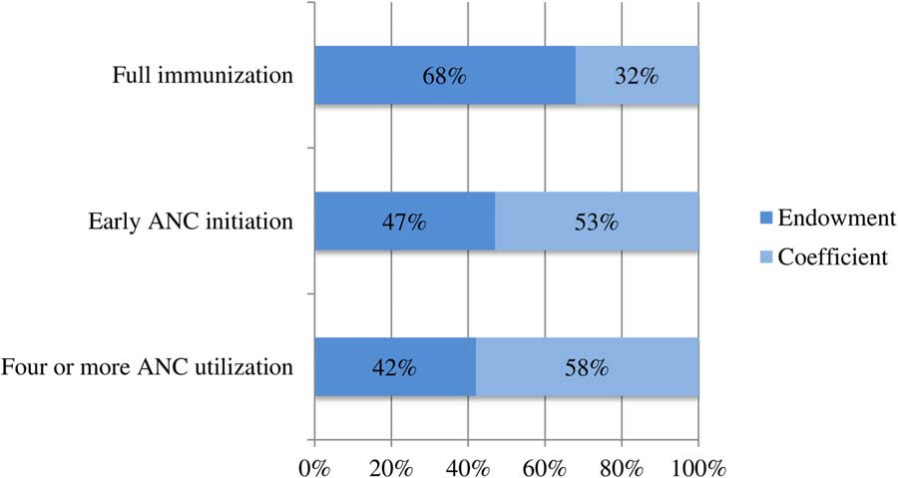


instead of:


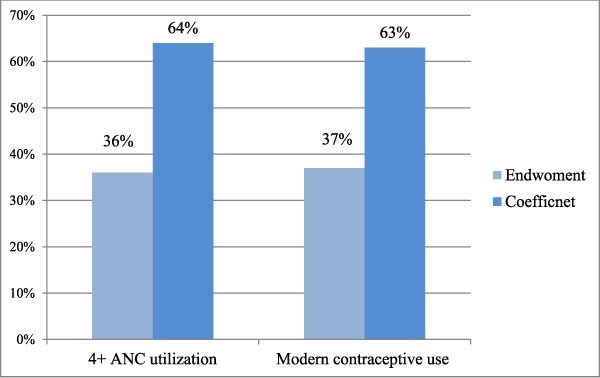


The **Study Funding** section text is emended to read: “This research was funded by the Gates Foundation through the Global Digital Health Exemplars project (Grant No. 142213).” instead of “No funding was received for this study.”

